# ﻿*Townsendialemhiensis* (Asteraceae, Astereae): A narrowly endemic new species from Idaho, USA

**DOI:** 10.3897/phytokeys.193.76365

**Published:** 2022-03-22

**Authors:** Christopher Lee, Curtis R. Björk, Jeannette Whitton

**Affiliations:** 1 School of Biological Sciences, Monash University, 25 Rainforest Walk, Clayton, Victoria, 3800 Australia; 2 Beaty Biodiversity Museum, University of British Columbia, 2212 Main Mall, Vancouver, BC, V6T 1Z4, Canada; 3 Department of Botany, Biodiversity Research Centre, and Beaty Biodiversity Museum, The University of British Columbia 3156 – 6270 6270 University Boulevard, Vancouver, BC, V6T 1Z4, Canada

**Keywords:** Apomixis, compositae, Flora of Idaho, Lemhi Valley

## Abstract

*Townsendialemhiensis* (Asteraceae) is described from the Lemhi Valley of east-central Idaho. From a genus with weak intrinsic isolating barriers, *T.lemhiensis* remains distinct apparently due to apomixis and to its isolation and habitat specialization on spatially limited occurrences of ashy white soils in the Lemhi Valley. Despite similarities to *T.spathulata*, this new species differs in its persistent pappus, fewer series of phyllaries and sericeous rather than long woolly hairs.

## ﻿Introduction

*Townsendia* Hook. includes about 29 recognized species distributed predominantly throughout the Rocky Mountain and Great Basin regions of western North America (Larsen 1927; Beaman 1957; Shultz and Holmgren 1980). Species of *Townsendia* are generally characterized by showy inflorescences nestled in a dense rosette of leaves or on short stems; and by a pappus of short bristles. Many species also have strong edaphic affinities that delimit their distribution (Lowrey and Knight 1994). Six species of *Townsendia* are known to occur in Idaho (*T.condensata* Parry, 1874, *T.florifera* (Hook.) A.Gray, 1880, *T.hookeri* Beaman, 1957, *T.leptotes* (A.Gray) Osterh., 1908, *T.montana* M.E.Jones, 1895, and *T.parryi* D.C.Eaton, 1874), yet none of these species approach the combination of characteristics present in *T.lemhiensis*.

## ﻿Methods

### ﻿Field methods

Specimens were initially collected during cursory exploration by Curtis Björk on May 20^th^, 2012. Curtis Björk further sampled the area in 2013, and two additional populations were found. In 2018, Chris Lee and Jeannette Whitton used localities provided by Curtis Björk to collect additional samples. We also used satellite imagery of nearby areas to identify potential habitat for *T.lemhiensis*, and these areas were targeted for additional surveying. Several voucher specimens were taken during each site visit, and dried between newspaper and cushioned cardboard in a plant press. Characters were measured directly from herbarium specimens. Herbaria acronyms follow Index Herbariorum (Thiers 2016).

### ﻿Pollen counts

We removed 2–3 florets from individual plants and placed them in a 200 μL microtube. After adding approximately 20 μL of lactophenol blue stain to each tube, we vortexed tubes for 20–30 seconds to release pollen into suspension. Slides were prepared using the resulting suspension, and viewed under a standard light microscope. This procedure was repeated multiple times on various specimens but yielded no pollen grains, thus no pollen counts or measurements could be completed. We later examined disk florets from individuals of all known populations under a dissecting microscope, and found that stamens were abortive and evidently non-functional; no anthers were observed.

## ﻿Taxonomic treatment

### ﻿ Townsendia lemhiensis

Taxon classificationPlantaeAsteralesAsteraceae

C.Lee, Björk & Whitton sp. nov.

6FF937BD-7D70-5EB6-A5CA-8C31FFE9F97E

urn:lsid:ipni.org:names:77296133-1

#### Holotype.

**Figure 1.**
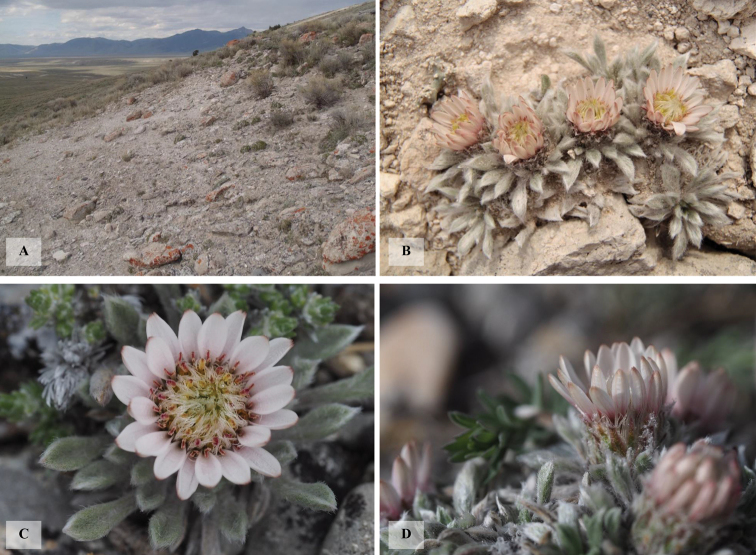
**A** habitat of the type locality of *Townsendialemhiensis***B** habit of *Townsendialemhiensis* at the type locality **C** close-up of *Townsendialemhiensis* capitulum **D** close-up of *Townsendialemhiensis* phyllaries. Photos A-B by Björk; Photos C-D by Whitton.

*Björk 29248* (UBC V252324) 20 May 2012. USA, Idaho, Lemhi County, 18 Mile Wilderness Area, ca. 24 km SSE of Leadore, 44.44806°N, 113.17222°W, on dry ashy white soil on slope (7368 ft) in sagebrush steppe (Fig. 1A). ***Paratypes***: *Björk 30627* (UBC V252325) 14 May 2013. USA, Idaho, Lemhi County, 18 Mile Wilderness Area, on a ridge crest near the middle road, 44.46707°N, 113.22570°W, on ashy white slope, sparsely vegetated, soil derived from tuff conglomerates, in upper elevation (6968 ft) sagebrush steppe in intermontane valley; *Björk 30772* (UBC V252326) 21 May 2013. USA, Idaho, Lemhi County, 18 Mile Wilderness Area, south of McFarland Road, 44.48000°N, 113.20333°W, on ashy white flats and slopes, sparsely vegetated, in upper elevation (6857 ft) sagebrush steppe in intermontane valley; *Whitton 252* (UBC V252322) 13 May 2018. USA, Idaho, Lemhi County, 18 Mile Wilderness Area, 44.47293°N, 113.20670°W, on open hillside (7355 ft) with sparse sagebrush and antelope brush on grey-brown rocky substrate; *Whitton 256* (UBC V252323) 14 May 2018. USA, Idaho, Lemhi County, 18 Mile Wilderness Area, 44.44530°N, 113.17541°W, on exposed rocky slope (7371 ft), in areas of grey-white substrate, with bunchgrass, and antelope brush.

Ab *Townsendiaspathulata* pappi ad maturitatem persistens, series phyllariorum pauciores, pilis caulibus sericeus non villosus, folia non carnosa differt.

#### Description.

**Figure 2.**
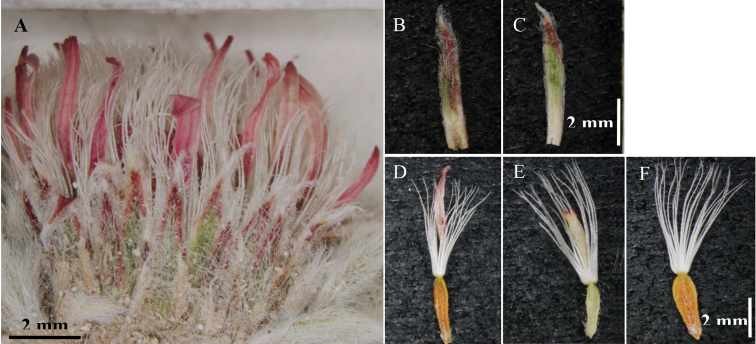
**A** capitulum **B** outer involucral bract **C** inner involucral bract **D** ray floret **E** disc floret **F** mature disc cypsela. Photos by Spencer Goyette (UBC Herbarium).

Plants perennial, 25–30 mm tall, rosette forming, rosettes 8–17 mm wide, solitary or (more often) arising from a few-branched caudex; stems essentially absent, or when visible, then villosulous; leaves 4–14 × 2 mm, narrowly oblanceolate to narrowly elliptic-oblanceolate, silvery, moderately sericeous but not villous, apex acute; capitula one per fertile rosette, sessile or nearly so; involucre campanulate, 9–10 mm wide, phyllaries appressed, narrowly elliptic-lanceolate, 6–7 × 1 mm, graduated, in 2–3 series, reddish brown, sericeous, apex acute to acuminate; ray florets 15–24, pistillate; ray corollas true ray, 7–8 mm long, surpassing pappus bristles, light brownish pink or whitish and tipped in pink, slightly darker abaxially, aglandular; disc florets 24–30, functionally pistillate; disc corollas tubular, 4 mm long, shortly surpassed by the pappus bristles, yellow, aglandular; stamens reduced to strap-like staminodes, anthers and pollen absent; cypselae 3 × 1 mm, oblanceolate and compressed, with glochidiate hairs; pappus bristles 23–26 in disc florets, 14–20 in ray florets, 6–7 mm long, white, barbellate, persistent. (Figs 1B–D, 2A–F).

#### Etymology.

*Townsendialemhiensis* is named after the Lemhi Valley, Idaho, where individuals of this species were first noticed by Curtis Björk.

#### Distribution and habitat.

*Townsendialemhiensis* is known only from the Lemhi Valley in east-central Idaho, which is situated within a region known for its numerous geographically endemic plants (Moseley and Conley 1989; Moseley et al. 1990; Ertter and Moseley 1992). The species has been found in three populations, within an area of 4.9 km^2^.

*Townsendialemhiensis* grows on ashy white slopes of eroded rhyolite tuff. These slopes of powdery soils and friable rock are sparsely vegetated, forming edaphic islands of open ground within a more densely vegetated surrounding matrix of sagebrush steppe. Numerous other plants having narrow geographical ranges occupy similar ashy slopes elsewhere in dry interior regions of western North America (Grimes 1984; Reveal and Björk 2004; Brown and Mansfield 2017). The Lemhi Valley is lined on the east and west by alpine ridges, and the valley floor sits at a high elevation, making it cooler than the sagebrush steppes both further south on the adjacent Snake River Plains and further north in the Salmon-Challis Valleys region.

Associated species growing with *Townsendialemhiensis* on the ashy slopes include: *Artemisiafrigida* Willd., 1838, Artemisiatridentatasubsp.wyomingensis Beetle & A.L.Young, 1965, *Chaenactisdouglasii* Hook. & Arn., 1839, *Comandrapallida* A.DC., 1857, *Cymopterusbipinnatus* S.Wats., 1885, *Elymuslanceolatus* (Scribn. and J.G.Sm.) Gould, 1949, *Eriogonummancum* Rydb., 1917, *Eriogonumsoliceps* Reveal & Björk, 2004, Ipomopsisspicatasubsp.orchidacea (Brand) D.Wilken & R.L.Hartm., 1991, Linumlewisiivar.alpicola Jeps., 1936, *Oreocaryahumilis* Greene, 1896, *Packeracana* (Hook.) W.A.Weber & Á.Löve, 1981, *Penstemonhumilis* Nutt. ex A.Gray, 1862, *Phloxmuscoides* Nutt., 1831, *Stenotusacaulis* (Nutt.) Nutt., 1840, *Townsendiahookeri* Beaman, 1957, *T.leptotes* (A.Gray) Osterh., 1908, and *T.parryi* D.C.Eaton, 1874.

#### Phenology.

We observed *T.lemhiensis* in flower in mid-May over three years (2012, 2013, 2018). At this time, some individuals had some capitula in bud, and others had mature seeds or open flowers. On this basis, we describe flowering as likely occurring throughout May, and seedset through late May or possibly into June. Further studies are needed to document the timing of bud formation, and potential variation in flowering and fruiting phenology. Another early-flowering species, *Townsendiahookeri*, co-occurs with *T.lemhiensis*, and has been observed to set buds in fall that open soon after snow melt (Lee and Whitton, personal observation). Given the early flowering of *T.lemhiensis* and the presence of snow patches persisting in surrounding areas, we suspect fall bud set may also occur in this species. Co-occurring species of *Townsendia* were found on site in bud (*T.parryi*) and late-bud (*T.leptotes* and *T.hookeri*).

## ﻿Discussion

*Townsendialemhiensis* is most similar in morphology to *T.spathulata* Nutt., which shares a generally hairy appearance. However, *T.lemhiensis* is morphologically distinct because its leaves are narrowly oblanceolate with short dense pubescence, instead of the fleshy, spathulate leaves covered in long, tangled hairs in *T.spathulata*. Also, the cypselae of *T.lemhiensis* have persistent pappi rather than the rare characteristic (in *Townsendia*) of deciduous pappi found only in *T.spathulata*, *T.microcephala* and *T.condensata* (Beaman 1957; Dorn 1992). Its capitulum is protected by fewer phyllary series (2–3), rather than 3–4 phyllary series found in *T.spathulata*.

**Figure 3.**
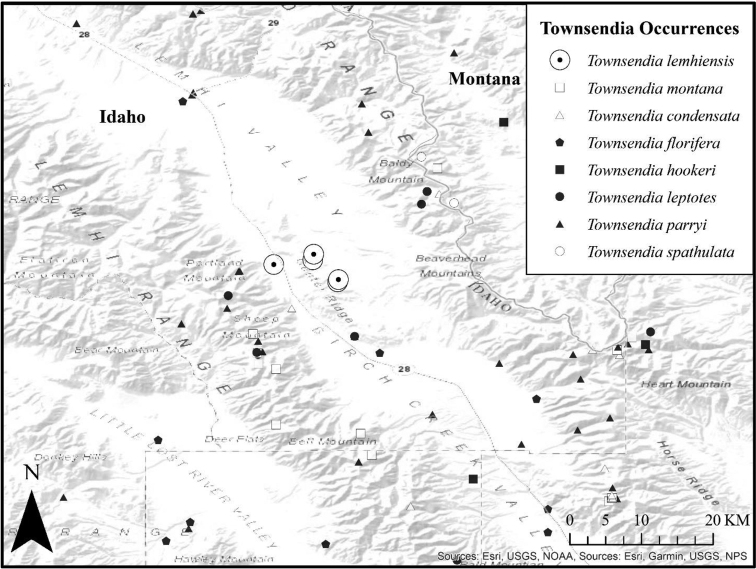
Occurrence records of *Townsendialemhiensis* and other nearby *Townsendia* spp. (ArcMap v.10.3.1) (Consortium of Pacific Northwest Herbaria 2022)

*Townsendialemhiensis* is morphologically distinct from the seven other species of *Townsendia* known from this region. We include *T.spathulata* here, because of its similarity to *T.lemhiensis*, and because it has been documented in the Beaverhead range overlooking the Lemhi Valley, Idaho (>9000 ft) at the border with Montana (Fig. 3). The seven species of *Townsendia* in Idaho and adjacent Montana can be distinguished using the taxonomic key below. Four of these species tend to occur at higher elevations (*T.condensata*, *T.leptotes*, *T.spathulata* and *T.montana*), while *T.florifera* is often at lower elevations than *T.lemhiensis*, and is associated with basalt. We found *T.parryi*, *T.leptotes* and *T.hookeri* co-occurring with *T.lemhiensis*, but these species were in bud, while *Townsendialemhiensis* was in full flower. The nearby locality of *T.spathulata* is somewhat unusual; more typically, *T.spathulata* inhabits the semi-arid plains of Wyoming and Montana along the eastern side of the Great Divide.

Although many *Townsendia* species occupy distinct geographical ranges and possess a multitude of unique character traits, Beaman (1957) describes them as lacking strong (intrinsic) genetic isolating barriers, based on crossing studies in the greenhouse, and on observed patterns of morphological intergradation in nature (Beaman 1954). While interbreeding is sometimes possible in cultivation, and likely occurs in nature, geographic isolation, phenological differences, and habitat differentiation, especially edaphic specialization, contribute to maintaining species boundaries in this genus. The species recognized by Beaman all include sexually reproducing populations, but apomictic populations also occur in a number of *Townsendia* species (Beaman 1954, 1957). Sexual populations are diploid and outcrossing, while known apomicts are triploid or tetraploid (Beaman 1954). Sexual and apomictic forms typically have distinct (sometimes overlapping) distributions, and provide classic examples of geographical parthenogenesis (Bierzychudek 1985). Apomixis also contributes to maintaining species boundaries, because hybridization involving apomicts is not likely. As a result, hybrids will not necessarily be produced, even where multiple *Townsendia* species co-occur.

Although direct genetic or experimental evidence of apomixis is not available for *T.lemhiensis*, no pollen was detected on florets sampled from our collections, which strongly suggests that these populations are apomictic. As a result, despite the physical proximity of *T.lemhiensis* to *T.hookeri*, *T.leptotes*, and *T.parryi*, hybridization is unlikely given that in this region, all four species are likely apomicts (Beaman 1957; Thompson and Whitton 2006). We note that while it is uncommon for species in *Townsendia* to be recognized based solely on apomictic populations, to date, apomicts have generally fit within the boundaries of morphological descriptions of known sexual populations. In this case, *T.lemhiensis* has no known sexual populations; whether these are undiscovered or extinct, or whether *T.lemhiensis* is of hybrid origin is not known.

The first author examined the majority of *Townsendia* specimens from UBC, UAC, SASK, RM, CS, and UNLV from 2008–2014, and the second author examined all Asteraceae at ID in 2002, and did not find any specimens of *Townsendialemhiensis*. Additionally, from 2008–2014, the first author undertook targeted searches for all species of *Townsendia* throughout their range in WA, ID, MT, WY, CO, NV, NM and CA, and no similar populations were encountered.

Despite the second author’s searches in apparently suitable habitats throughout the Lemhi Valley and adjacent valleys in Idaho and Montana in the years 1999–2013, only the three reported populations have been located. Thus it appears that *T.lemhiensis* is a high priority for conservation. All known occurrences of *T.lemhiensis* are situated on public land administered by the Bureau of Land Management. Hence, the landscape surrounding this species is somewhat protected from threats, but such a small, rare species may still undergo population reduction from factors such as trampling by cattle or recreationists, invasive exotic plant species, or from climate change that could cause higher frequency and severity of drought and excessive heat events.

### ﻿Key to *Townsendia* of Idaho and adjacent Montana

**Table:** 

1a	Lax-growing plants with elongated stems, many internodes > 2 mm long, plants often lacking sterile rosettes when flowering	**2**
2a	Stems erect or sub-erect; longest phyllaries > 9 mm long	**3**
3a	Rays purplish; longest phyllaries mostly > 10 mm long; stems erect, unbranched	***T.parryi***
3b	Rays white or pink; longest phyllaries mostly < 10 mm long; stems sub-erect, often branched	***T.florifera***
2b	Stems decumbent; longest phyllaries up to 9 mm long	**4**
4a	Stems gray-white, obscured by dense hairs	***T.incana***
4b	Stems lightly to moderately strigose	***T.strigosa***
1b	Compact plants, stems scarcely elongated, internodes < 2 mm long; sterile rosettes commonly present	**5**
5a	Plants with abundant spreading hairs	**6**
6a	Pappus persistent; leaf hairs sericeous	***T.lemhiensis***
6b	Pappus easily breaking away from the cypselae at maturity; leaf hairs villous	**7**
7a	Phyllaries > 40, the longest ones > 9 mm long, involucres > 12 mm long	***T.condensata***
7b	Phyllaries < 40, the longest ones < 9 mm long, involucres < 12 mm long	***T.spathulata***
5b	Plants with appressed hairs only, or spreading hairs few and inconspicuous	**8**
8a	Phyllaries 2–5 × long as wide; rays purplish, often glandular abaxially	**9**
9a	Phyllaries acute to acuminate; ray laminae 5–8 mm long	***T.leptotes***
9b	Phyllaries blunt; ray laminae 7–16 mm long	***T.montana***
8b	Phyllaries at least 6 × long as wide; rays white to pink, glabrous	**10**
10a	Longest phyllaries > 12 mm long and > 1.5 mm wide; disc pappi 6.5–11+ mm long; leaf midvein apparent	***T.exscapa***
10b	Longest phyllaries < 12 mm long and ca. 1 mm wide; disc pappi 4–6(-7.5) mm long; leaf midvein obscure	***T.hookeri***

## Supplementary Material

XML Treatment for Townsendia lemhiensis

## Supplementary materials

Supplementary material 1 *Townsendialemhiensis* occurence records Data type Occurence. Explanation note Type and paratype specimens collected of *Townsendialemhiensis*. https://binary.pensoft.net/file/661634This dataset is made available under the Open Database License (http://opendatacommons.org/licenses/odbl/1.0/). The Open Database License (ODbL) is a license agreement intended to allow users to freely share, modify, and use this Dataset while maintaining this same freedom for others, provided that the original source and author(s) are credited.Christopher Lee, Curtis R. Björk, Jeannette Whitton
